# Deep Sequencing Analysis of HBV Genotype Shift and Correlation with Antiviral Efficiency during Adefovir Dipivoxil Therapy

**DOI:** 10.1371/journal.pone.0131337

**Published:** 2015-06-25

**Authors:** Yuwei Wang, Xuefeng Shan, Zhi Liang, Youlan Shan, Wenxiang Huang, Dazhi Zhang, Aizhong Zen, Xin Zhou, Yao Zhao, Xuyang Gong, Ge Xu, Xiuyu Zhang, Juan Chen, Ailong Huang

**Affiliations:** 1 Key Laboratory of Molecular Biology on Infectious Diseases, Ministry of Education, Chongqing Medical University, Chongqing, China; 2 School of Life Sciences, University of Science and Technology of China, Hefei, Anhui, China; 3 Department of Infectious Diseases, the Second Affiliated Hospital, Chongqing Medical University, Chongqing, China; 4 Department of Infectious Diseases, the First Affiliated Hospital, Chongqing Medical University, Chongqing, China; 5 Pediatric Research Institute, Children's Hospital of Chongqing Medical University, Chongqing, China; 6 Department of Laboratory Medicine, Chongqing Hospital of Traditional Chinese Medicine, Chongqing, China; 7 Department of Pharmacy, the First Affiliated Hospital, Chongqing Medical University, Chongqing, China; Indiana University, UNITED STATES

## Abstract

**Background:**

Viral genotype shift in chronic hepatitis B (CHB) patients during antiviral therapy has been reported, but the underlying mechanism remains elusive.

**Methods:**

38 CHB patients treated with ADV for one year were selected for studying genotype shift by both deep sequencing and Sanger sequencing method.

**Results:**

Sanger sequencing method found that 7.9% patients showed mixed genotype before ADV therapy. In contrast, all 38 patients showed mixed genotype before ADV treatment by deep sequencing. 95.5% mixed genotype rate was also obtained from additional 200 treatment-naïve CHB patients. Of the 13 patients with genotype shift, the fraction of the minor genotype in 5 patients (38%) increased gradually during the course of ADV treatment. Furthermore, responses to ADV and HBeAg seroconversion were associated with the high rate of genotype shift, suggesting drug and immune pressure may be key factors to induce genotype shift. Interestingly, patients with genotype C had a significantly higher rate of genotype shift than genotype B. In genotype shift group, ADV treatment induced a marked enhancement of genotype B ratio accompanied by a reduction of genotype C ratio, suggesting genotype C may be more sensitive to ADV than genotype B. Moreover, patients with dominant genotype C may have a better therapeutic effect. Finally, genotype shifts was correlated with clinical improvement in terms of ALT.

**Conclusions:**

Our findings provided a rational explanation for genotype shift among ADV-treated CHB patients. The genotype and genotype shift might be associated with antiviral efficiency.

## Introduction

The prevalence of chronic Hepatitis B virus (HBV) infection varies greatly in different parts of the world [1,2]. HBV has been classified into ten different genotypes (from A to J) based on the divergence of over 8% of the entire HBV genome sequence [3–5]. It has been reported that HBV genotype influences the outcome of HBV infection as well as the response to antiviral therapy [6–9].

HBV genotype shift has been reported previously in 18% to 32% of chronic hepatitis B (CHB) patients during antiviral therapy [10–15]. Recently, in a longitudinal study, Jardi et al observed a 53% genotype shift rate in Spanish patients under antiviral therapy using INNO-LiPA method [16]. Therefore, HBV genotype shift might be a common phenomenon in CHB patients during antiviral therapy. However, the underlying molecular mechanism of genotype shift still remains elusive.

So far, there are several possible explanations for HBV genotype shift in CHB patients undergoing antiviral therapy. Superinfection with another genotype during the treatment may result in genotype shift. Alternatively, the patient may initially carry a mixed infection with minor genotype undetectable by the current methods but the dominant genotype changes under the selection pressure from antiviral therapy. Although the last possibility of genotype shift has been widely proposed by many investigators, no solid supporting data are available experimentally [10–14]. This study aimed to investigate the mechanism of genotype shift in Chinese CHB patients and the influence of the HBV genotype on the disease course and treatment outcome, which was poorly documented.

Interestingly, the mixed infection rates of HBV genotypes vary significantly with different methods used. For example, in our previous study, we observed the rate of mixed genotype infection increased to 30% based on a more sensitive GQ-PCR method, while only 17% were detected with Sanger sequencing from the same sample pools [17]. However, with INNO-LiPA method, mixed infections rate reached 22% in 103 CHB patients [16]. With the development of the next generation sequencing technology, the rate of mixed infections of HBV among CHB patients might be higher than expected.

In this study, we demonstrate the successful application of deep sequencing technology to investigate the possible mechanism of genotype shift and its association with antiviral efficiency in CHB patients receiving antiviral therapy. We found high prevalence of mixed genotype infection before ADV treatment in CHB patients and the different sensitivity of distinct HBV genotypes to drug selection pressure might contribute to genotype shifts. Furthermore, we discovered that genotype C was more sensitive to ADV than genotype B and patients with dominant genotype C may have a better clinical improvement. Our data suggest that determination of genotype distributions of CHB patients using deep sequencing technology can be much accurate for diagnosis and monitoring during antiviral therapy and the different sensitivity of various genotypes to ADV therapy might provide a new strategy of antiviral therapy.

## Methods

### Patients and specimens

38 CHB patients treated with adefovir dipivoxil (ADV) for 48 weeks were selected based on the following criteria: HBV load greater than 1x10^5^ copies/ml, ALT higher than 1.3 fold of the upper normal limit (UNL) and positive for HBeAg. Patients with liver cirrhosis, alcohol/drug abuse and HCV/HDV/HIV co-infection were excluded. Serum specimens were obtained from the 38 CHB patients who underwent ADV monotherapy from 2004 to 2005 in the First Affiliated Hospital of Chongqing Medical University. Written informed consent was obtained from all patients, and the study protocol was approved by the Ethical Committee of the Chongqing Medical University in accordance with the Declaration of Helsinki.

### Establishment of genotyping analysis by deep-sequencing based on short sequence windows

The genotyping methodology was described detailedly in S1 Fig. Validation of genotyping analysis by deep-sequencing was described in S1 File and S2 Fig.

### Direct and clonal sequencing for HBV genotype analysis

The method was detailed in S1 File.

### Massively parallel deep sequencing for HBV genotyping

The method was described in detail in S1 File.

### Statistical analysis

Statistical analysis was performed with ANOVA method to the obtained data and with Chi square test for comparisons between two groups. Results were considered statistically significant when *p*<0.05.

## Results

### Clinical laboratory data of the study population and genotype distribution according to direct sequencing method

Thirty-eight treatment-naïve patients with CHB were enrolled in the present study. All of the 32 male and 6 female patients were of hepatitis B e antigen (HBeAg) positive before ADV treatment with a median age of 30.3 (range: 19–52 years old). The clinical parameters of these HBV patients are summarized in Table A in S1 File. The genotype distribution was first determined by conventional direct sequencing method. Before ADV treatment, genotype B, genotype C and mixed B+C genotypes were detected in 25 (65.8%), 11 (28.9%) and 2 (5.3%) patients, respectively. After ADV treatment, genotype B, genotype C and mixed B+C genotypes were detected in 27 (71.1%), 4 (10.5%) and 7 (18.4%) patients, respectively. Genotype shifts were observed in 13 patients after ADV treatment (Table 1).

**Table 1:** HBV genotype, ALT, HBeAg and HBV-DNA levels of 38 patients before and after ADV therapy.

Case number		ADV	ADV
		before	after
1	Genotype	B	B
	ALT	184	185
	HBV-DNA	8.8	7.9
	HBeAg	+	+
2	Genotype	B	B
	ALT	104	23
	HBV-DNA	7.6	5.5
	HBeAg	+	-
3	Genotype	B	B
	ALT	94	17
	HBV-DNA	9.8	5.5
	HBeAg	+	-
4	Genotype	B	B
	ALT	80	54
	HBV-DNA	9.9	7.8
	HBeAg	+	+
5	Genotype	C	C
	ALT	157	35
	HBV-DNA	9.4	5.5
	HBeAg	+	+
6	Genotype	B	B
	ALT	95	451
	HBV-DNA	10.4	8.3
	HBeAg	+	+
7	Genotype	B	B
	ALT	235	16
	HBV-DNA	10.3	5.1
	HBeAg	+	-
8	Genotype	C	B
	ALT	290	72
	HBV-DNA	8.6	5.7
	HBeAg	+	-
9	Genotype	C	B/C
	ALT	203	35
	HBV-DNA	8.5	5.5
	HBeAg	+	+
10	Genotype	B	B
	ALT	107	45
	HBV-DNA	8.9	6.3
	HBeAg	+	+
11	Genotype	C	B/C
	ALT	260	47
	HBV-DNA	9.8	5.5
	HBeAg	+	-
12	Genotype	B	B
	ALT	611	86
	HBV-DNA	9	6.2
	HBeAg	+	+
13	Genotype	B	B
	ALT	87	103
	HBV-DNA	9.1	5.8
	HBeAg	+	-
14	Genotype	C	B/C
	ALT	300	31
	HBV-DNA	8.3	5.1
	HBeAg	+	+
15	Genotype	B	B
	ALT	136	71
	HBV-DNA	9.2	7.2
	HBeAg	+	+
16	Genotype	B	B/C
	ALT	351	42
	HBV-DNA	7.3	3
	HBeAg	+	-
17	Genotype	B	C
	ALT	151	26
	HBV-DNA	8.7	5.1
	HBeAg	+	-
18	Genotype	B	B/C
	ALT	161	18
	HBV-DNA	9.9	4.7
	HBeAg	+	+
19	Genotype	B/C	B
	ALT	50	29
	HBV-DNA	8.4	6.2
	HBeAg	+	-
20	Genotype	B	B
	ALT	193	31
	HBV-DNA	8.5	6
	HBeAg	+	-
21	Genotype	B/C	B
	ALT	235	18
	HBV-DNA	7.7	3.9
	HBeAg	+	-
22	Genotype	B	B
	ALT	59	19
	HBV-DNA	10.5	8.6
	HBeAg	+	+
23	Genotype	B	B
	ALT	130	120
	HBV-DNA	10.6	10.6
	HBeAg	+	+
24	Genotype	B	B
	ALT	104	24
	HBV-DNA	10.7	8.2
	HBeAg	+	-
25	Genotype	B	B
	ALT	63	68
	HBV-DNA	9.8	8.8
	HBeAg	+	+
26	Genotype	C	B/C
	ALT	73	24
	HBV-DNA	9.2	9.4
	HBeAg	+	+
27	Genotype	C	B
	ALT	145	53
	HBV-DNA	9.7	8.2
	HBeAg	+	-
28	Genotype	C	C
	ALT	282	58
	HBV-DNA	9.5	9.1
	HBeAg	+	-
29	Genotype	B	B
	ALT	54	33
	HBV-DNA	9.3	8.1
	HBeAg	+	+
30	Genotype	B	B
	ALT	252	242
	HBV-DNA	10.1	9.1
	HBeAg	+	+
31	Genotype	B	B
	ALT	98	114
	HBV-DNA	10.6	10.1
	HBeAg	+	+
32	Genotype	B	B
	ALT	45	49
	HBV-DNA	10.6	9.5
	HBeAg	+	+
33	Genotype	C	B/C
	ALT	62	67
	HBV-DNA	11	9.8
	HBeAg	+	+
34	Genotype	C	C
	ALT	74	29
	HBV-DNA	10.2	9
	HBeAg	+	-
35	Genotype	B	B
	ALT	161	35
	HBV-DNA	10.8	9.7
	HBeAg	+	-
36	Genotype	B	B
	ALT	72	46
	HBV-DNA	10.4	8.9
	HBeAg	+	+
37	Genotype	C	B
	ALT	342	19
	HBV-DNA	10.2	9.5
	HBeAg	+	-
38	Genotype	B	B
	ALT	50	38
	HBV-DNA	9.3	9.4
	HBeAg	+	-

Patients 3,7,13,16,17,19,20,24,37,38 with HBeAg seroclearance. Patients 2,8,11,21,27,28,34,35 with HBeAg seroconversion. ALT expressed as IU/L, HBVDNA expressed as log_10_copies/mL.

### Genotype distribution according to clonal sequencing and deep sequencing methods

**Fig 1:**
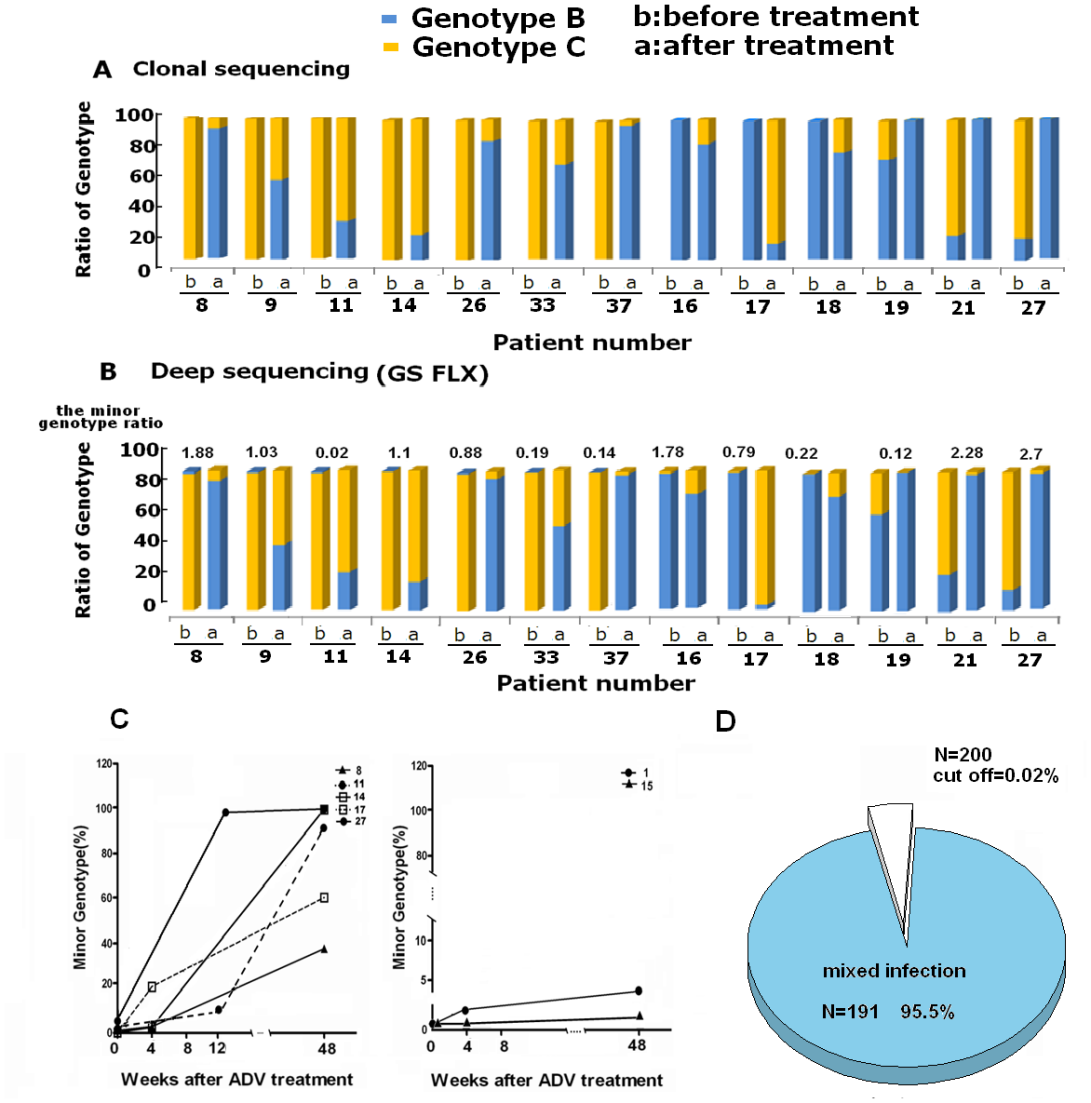
Prevalence of mixed genotype infection before ADV treatment in CHB patients. (A) Genotype distributions of 13 patients with genotype shift observed by clonal sequencing showed that 7 patients were genotype C, 3 patients were genotype B and 3 patients were mixed infection before drug treatment. (B) Genotype patterns observed by GS FLX revealed that all 13 patients were of mixed infection (B+C) before and after antiviral therapy. The ratios of minor genotype were on the top of figure, as determined by deep sequencing GS FLX. (C) The change of minor genotypes in course of ADV treatment. The genotype distributions in 13 patients were analyzed at week 0, week 4, week 12 and week 48 after ADV treatment. The alterations of minor genotype in 7 samples including 5 patients with genotype shift and 2 patients without genotype shift were presented. (D) The genotype distribution was analyzed in 200 CHB patients before ADV treatment by Solexa method. 95.5% patients showed mixed genotypes.

The genotype distribution and mixed infection rate were further determined by conventional cloning sequencing method. Before ADV treatment, genotype B was detected in 25 (65.8%), genotype C in 10 (26.3%) and mixed B+C genotypes in 3 patients (7.9%), respectively. The minor genotype ratios in the 3 patients with mixed B+C genotypes were 6.5%, 9.5% and 27.8% (Fig 1A). After ADV treatment, genotype B was detected in 25 (65.8%), genotype C in 3 (7.9%) and mixed B+C genotypes in 10 patients (26.3%), respectively.

Deep sequencing (GS FLX platform) was also applied to investigate the genotype distributions of the 38 patients. In contrast to clonal sequencing result, mixed B+C genotypes were present in all the patients before ADV treatment. The detailed genotype distributions of the 13 patients with genotype shift are summarized in Fig 1B and Table 2. The minor genotype ratio in these patients ranged from 0.02% to 2.7% before ADV therapy. The genotype change of the 13 patients was further analyzed at several key time points including week 0, week 4, week 12 and week 48. As shown in Fig 1C, the fraction of the minor genotype in 5 patients (38%) with genotype shift increased gradually during the course of ADV treatment, in contrast to the slight alterations of minor genotype in 2 patient without genotype shift (Fig 1C). The fact indicates that the minor genotype becomes progressively dominant during ADV treatment, which may eventually lead to genotype shift. Therefore, the mechanism underlying genotype shift may be the change of different genotype ratios driven by certain pressures.

**Table 2:** Genotypes distribution of the 13 patients with genotype shifts determined by clonal and deep sequencing.

Patients	Clonal sequencing (clones)					Deep sequencing (reads)				
	Total (clones)	B%	(clones)	C%	(clones)	Total (reads)	B%	(reads)	C%	(reads)
8 B	44	0	(0)	100	(44)	532	1.88	(10)	98.12	(522)
A	36	91.7	(33)	8.3	(3)	4240	92.02	(3902)	7.98	(338)
9 B	31	0	(0)	100	(31)	3802	1.03	(39)	98.97	(3763)
A	30	56.7	(17)	43.3	(13)	4758	47.23	(2247)	52.77	(2511)
11 B	32	0	(0)	100	(32)	4107	0.02	(1)	99.98	(4106)
A	32	18.8	(6)	81.2	(26)	3716	20.67	(768)	79.33	(2948)
14B	35	0	(0)	100	(35)	1539	1.1	(17)	98.9	(1522)
A	30	26.7	(8)	73.3	(22)	3041	27.39	(833)	72.61	(2208)
16B	32	100	(32)	0	(0)	1795	98.22	(1763)	1.78	(32)
A	34	82.4	(28)	17.6	(6)	3004	82.84	(2489)	17.16	(515)
17B	44	100	(44)	0	(0)	1772	99.21	(1758)	0.79	(14)
A	30	13.3	(4)	86.7	(26)	1259	3.14	(39)	96.86	(1220)
18B	35	100	(35)	0	(0)	1383	99.78	(1380)	0.22	(3)
A	31	77.4	(24)	22.6	(7)	3156	82.83	(2614)	17.17	(542)
19 B	36	72.2	(26)	27.8	(10)	1250	70.48	(881)	29.52	(369)
A	32	100	(32)	0	(0)	5198	99.88	(5192)	0.12	(6)
21B	31	6.5	(2)	93.5	(29)	2044	16.39	(335)	83.61	(1709)
A	31	100	(31)	0	(0)	1839	97.72	(1797)	2.28	(42)
26B	34	0	(0)	100	(34)	4545	0.88	(40)	99.12	(4505)
A	38	84.2	(32)	15.8	(6)	3707	94.36	(3498)	5.64	(209)
27 B	42	9.5	(4)	94.5	(38)	4006	5.32	(213)	94.68	(3793)
A	35	100	(35)	0	(0)	4216	97.3	(4102)	2.7	(114)
33B	33	0	(0)	100	(33)	4724	0.19	(9)	99.81	(4715)
A	41	68.3	(8)	31.7	(13)	5950	60.82	(3619)	39.18	(2331)
37 B	32	0	(0)	100	(32)	4205	0.14	(6)	99.86	(4199)
A	30	96.6	(29)	3.4	(1)	3428	97.78	(3352)	2.22	(76)

A refers to after treatment; B refers to before treatment.

In view of the discrepancies observed, additional 200 CHB patients were analyzed with the deep sequencing method (Solexa platform). A high rate up to 95.5% of mixed HBV genotypes was observed by Solexa method (Fig 1D). Mixed B+C, A+B+C and B+C+D genotypes were observed in 187, 3 and 1 patients, respectively. These data further confirmed the prevalence of mixed genotype infections in patients before ADV treatment and may be prerequisite for genotype shift.

### Genotype shifts correlated with selection pressure

**Fig 2:**
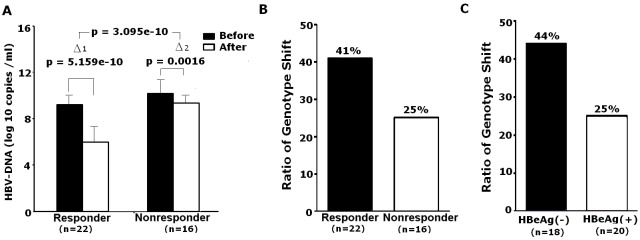
Genotype shifts correlated with selection pressure. (A) 38 patients were classified into the responder (HBV DNA load decreased by greater than 1x10^2^ copies /ml after ADV treatment) and non-responder groups (DNA load decreased by less than lx10^2^ copies /ml after ADV treatment). (B) Genotype shift was observed in 41% (9/22) of the responders and 25% (4/16) of non-responders, respectively. (C) The patients were divided into HBeAg-negative and HBeAg-positive group based on the level of HBeAg after drug treatment. Genotype shift was observed in 44% (8/18) of HBeAg-negative group and 25% (5/20) of HBeAg-positive group following the drug treatment.

To further investigate the underlying mechanism of genotype shift, association between genotype shift and selection pressure (including drug pressure and immune pressure) during antiviral therapy was analyzed. NAs inhibit HBV replication by targeting viral DNA polymerase, therefore, the change of HBV DNA load could reflect the response strength of HBV to drug selection pressure. Meanwhile, the change of serum HBeAg level or HBeAg seroconversion was used to indicate immune pressure strength of HBV as it functions to modulate immune response during chronic HBV infection. Based on serum HBV DNA level after ADV treatment, the 38 patients were classified into responder (HBV DNA load decreased by greater than 1x10^2^ copies/ml) and non-responder groups (DNA load decreased by less than lx10^2^ copies/ml) (Fig 2A). The response to ADV induced by drug selection pressure was positively correlated with the rate of genotype shift, (Fig 2B). Furthermore, the rate of genotype shift was much higher in patients losing HBeAg during therapy (44%) relative to those remaining HBeAg-positive (25%) (Fig 2C). In contrast, absence of genotype shift was often observed in HBeAg-positive poor responder (data not shown). Collectively, these data support the hypothesis that immune and drug pressure could induce genotype shift and different sensitivity of various genotypes to selection pressures may be the key factor contributing to genotype shift.

### HBV genotype C was more sensitive to ADV than genotype B

**Fig 3:**
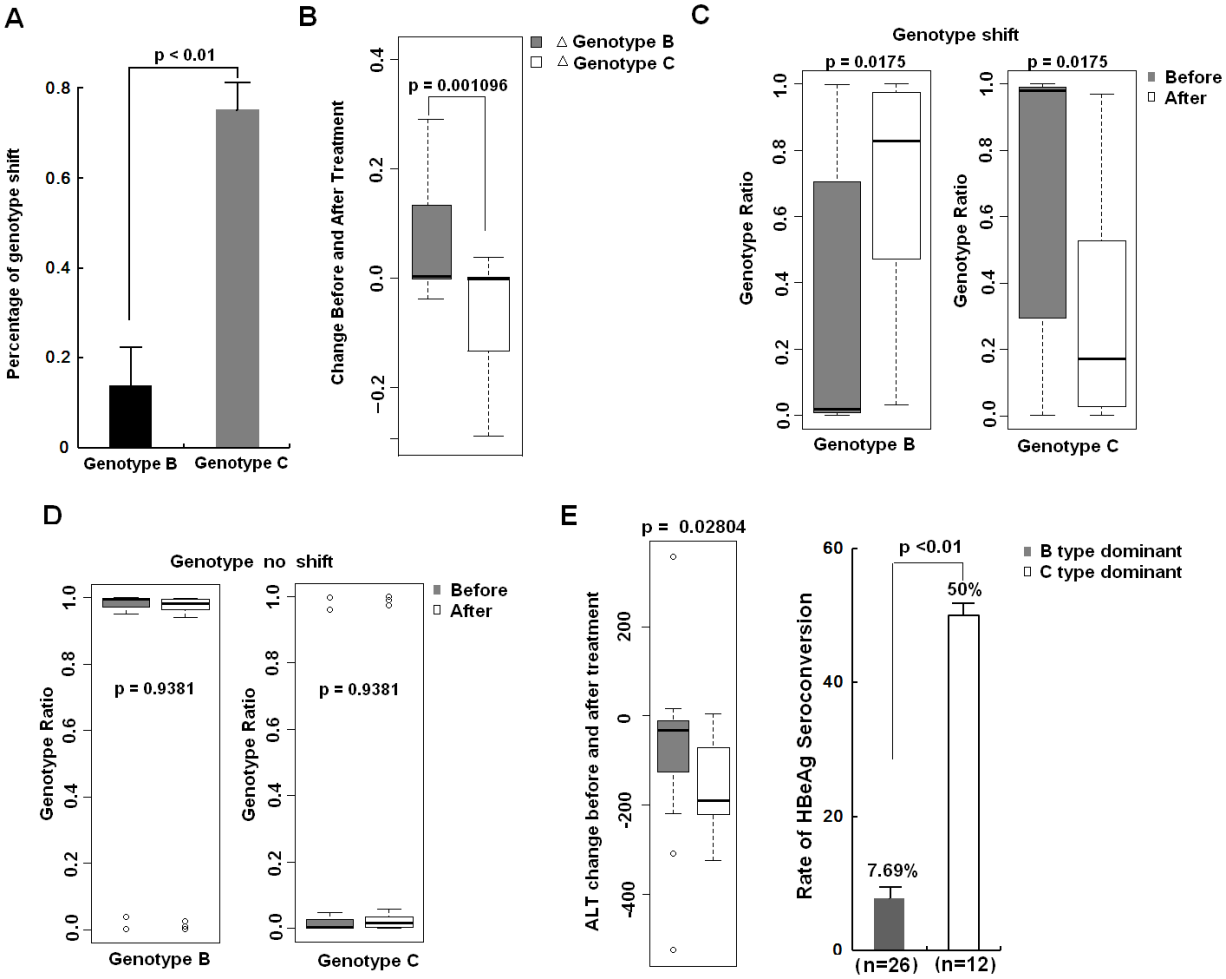
HBV genotype C was more sensitive to ADV than genotype B. (A) 38 patients were divided into genotype B group and genotype C group based on the genotype before ADV treatment analyzed by Sanger sequencing. Patients with genotype C showed higher rate of genotype shift than genotype B after ADV treatment. (B) The change of genotype B and C ratios in 38 patients before and after ADV treatment analyzed by deep sequencing method. ΔGenotype B = Genotype B ratio after ADV treatment- Genotype B ratio before ADV treatment; ΔGenotype C = Genotype C ratio after ADV treatment- Genotype C ratio before ADV treatment. (C) Genotype shift (GS) group showed increased ratio of genotype B accompanied by decreased ratio of genotype C after ADV treatment (*p* = 0.0175). (D) Group without genotype shift (NS) showed no significant difference of genotype B or C ratio after ADV treatment (*p* = 0.9381). (E) Comparison of change in ALT and the rate of HBeAg seroconversion after ADV therapy between patients with dominant genotype B and C. B, C, D and E, 38 patients were divided into genotype B group and genotype C group based on the genotype analyzed by deep sequencing method before ADV treatment.

Recently, HBV genotypes have been implicated in response to interferon therapy. Thus, we next determined the effects of HBV genotype on ADV response. According to the result of Sanger method, patients with genotype C had a significantly higher rate of genotype shift relative to patients with genotype B (75% vs. 15%; p<0.01) (Fig 3A), suggesting genotype C may be more sensitive to ADV treatment. Further analysis of the deep sequencing data of the 38 patients showed that the change of genotype B before and after ADV treatment was significantly different from genotype C (Fig 3B). Generally, the ratio of genotype B increased gradually in contrast to steady decline of the ratio of genotype C during ADV therapy. To further determine whether genotype was correlated with drug response, the 38 patients were divided into genotype shift group (N = 13) and no shift group (N = 25) and the change of genotype B and C before and after treatment in these two groups were analyzed, respectively. In genotype shift group, ADV treatment induced a marked enhancement of genotype B ratio accompanied by a robust reduction of genotype C ratio (Fig 3C). However, the change of genotype B or C ratio before and after ADV therapy did not differ significantly in group without genotype shift (Fig 3D). On the other hand, the reduction of ALT and rate of HBeAg serconversion after ADV therapy were more significant in patients with dominant genotype C before treatment compared with paitents with dominant genotype B (Fig 3E), suggesting patients with dominant genotype C may have a better therapeutic effect. Collectively, these data revealed that HBV genotype C may be more sensitive to ADV than genotype B, and therefore is more prone to be associated with genotype shift during ADV therapy.

### Correlations between genotype shift and clinical therapeutic parameters

**Fig 4:**
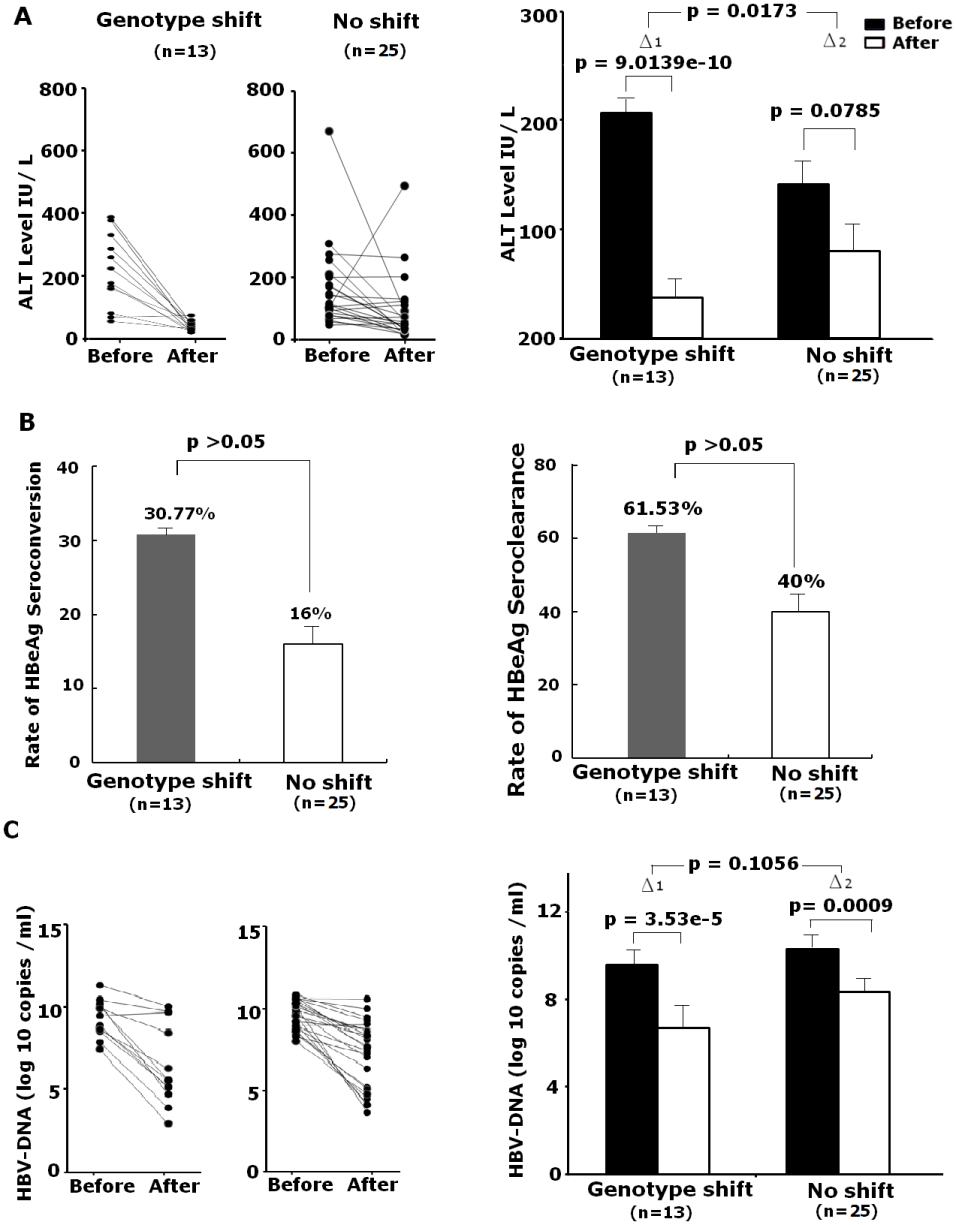
The correlation between genotype shift and clinical therapeutic parameters. (A) Left, change of ALT level after ADV treatment. Right, the decrease in ALT levels of the genotype shift (GS) group was significantly higher than group without genotype shift (NS) (*p* <0.05). (B) Left, comparison of HBeAg seroconversion after ADV treatment between the GS group and the NS group (*p* >0.05). Right, comparison of HBeAg seroclearance after ADV treatment between the GS group and the NS group(*p* >0.05). (C)Left, change of HBV DNA load after ADV treatment. Right, the reduction of HBV DNA load in GS group and NS group did not different significantly (*p* >0.05).

Three clinical parameters, including ALT, HBV DNA load and HBeAg level were used to assess clinical therapeutic effect. After ADV treatment, patients with genotype shift showed a more significant reduction of ALT (*p* = 0.0173) compared with patients without genotype shift (Fig 4A). However, the rate of HBeAg seroconversion or seroclearance and change of HBV DNA load in patients with and without genotype shift did not differ significantly (*p*>0.05) (Fig 4B and 4C). Together, these data suggested that genotype shift was correlated with clinical improvement in terms of ALT.

## Discussion

In this study, a methodology based on deep sequencing technology was developed for the comprehensive and efficient genotype analysis of HBV from a large number of clinical samples. Our objective was to investigate the possible mechanism of HBV genotype shift in CHB patients under ADV therapy and its correlation with antiviral efficiency.

The reported genotype shift rates ranged from 18% to 32% depending on the source of clinical specimens and the methods used [10–15]. Recently, Jardi et al found that HBV genotype shifts were observed in 9 (53%) of 17 antiviral-treated patients from Spain using INNO-LiPA method and only 2 (22%) out of 9 patients of genotype shifts were mixed infection at baseline. Therefore, the hypothesis of mixed infection contributing to genotype shift could not be supported directly by Jardi’s findings due to small sample size and the low rate of mixed infection. In our study, both Sanger and deep sequencing methods were used for genotype analysis in 38 ADV-treated CHB patients from China. According to the Sanger method, 3 patients (7.9%) had mixed genotypes before treatment and 13 (34%) of 38 patients showed genotype shifts during treatment. However, all these 38 patients were shown to be of mixed infection with multiple genotypes by deep sequencing method. Furthermore, the high prevalence of mixed infection was further confirmed from another set of 200 CHB patients by deep sequencing. Our data revealed that deep sequencing-based genotyping methods are far more sensitive than conventional method previously used[18–21]. In addition, two different selected windows along the full HBV genome gave identical results of genotyping with respect to the same CHB patient, indicating that the obtained results from deep sequencing are reliable and comparable to the results derived from clonal sequencing. Therefore, we believe that the established deep sequencing methodology is a powerful tool for obtaining more profound insight into the study of genotype shifts and genotype-specific drug response during antiviral therapy.

Due to the high sensitivity of deep sequencing (with minor genotype detection limit of 0.02% in this study), the hidden minor genotypes in mixed infections could be detected before drug treatment. Based on the above analysis, it is reasonable to conclude that the detected genotype shift probably resulted from the initial existence of mixed genotypes. Since no single genotype conversion from B to C or from C to B was observed by deep sequencing in any of our 38 study cases, meanwhile the divergence of ancestral HBV sequence into 8 genotypes is a long evolutionary process which can never be reproduced during a single patient’s lifespan, the mechanism of gene mutation might be excluded in this study. In a study from Germany involving 13 children suffering from chronic liver disease, HBV genotype conversion from types D to A was observed in 3 babies of 4 month old[11]. These findings suggested D/A genotype shift within such a short period could only be the result of initial mixed infections rather than gene mutation or superinfection. Furthermore, a dominant genotype change from C to B during the early antiviral therapy (day 22 post-treatment) also supported that genotype shift could only be caused by the initial mixed infections[14].

The genotype shifts associated with selection pressure during the treatment were also investigated. The results of both direct sequencing and clonal sequencing method revealed that rate of mixed infection in 13 patients with genotype shift increased significantly after ADV therapy. Interestingly, enhancement of degree of mixed infection was also found after ADV treatment by deep sequencing GS FLX method in majority of 13 patients with genotype shifts. As control, 5 treatment-naive CHB patients with interval of more than one year showed no genotype shifts (data not shown). These data suggested that drug pressure may be an important factor to induce genotype shift. This hypothesis was further supported by the association analysis between genotype shift and selection pressure. The drug responders were found to have significantly higher rates of genotype shifts as compared to non-responders. Further comparisons between genotype shifts and the HBeAg seroclearance clearly demonstrated that the HBeAg seroclearance was also correlated with the high incidence of genotype shift. These results indicated that selection pressure (drug or immunologic) may be the external conditions inducing HBV genotype shift. Concordantly, Jardi et al also reported that genotype changes only occurred in CHB patients under antiviral therapy. Compared to the three years study by Jardi et al, our one year study appeared to have the lower rate of genotype shift (53% vs.34%). Among a group of 33 HIV/HBV co-infected patients, 3–6 months of antiviral therapy also resulted in 18% of the HBV genotype shift[15]. It is likely that the incidence of genotype shift could be increased with the duration of NAs-related drug treatment, despite different patient sources in these studies. Since NAs is the current first-line antiviral therapy drug for CHB[22–24], genotype shift during treatment may become a common phenomenon with the widespread use of NAs.

Based on the literature, there are still no consensus regarding the correlations between genotypes and differential sensitivity of nucleotide analogues. Reports by Liu and Mirandola seemed to suggest the differential response of genotypes to different NAs[25,26]. Jardi results suggest that genotype A strains were likely present in an extremely low proportion in pre-treatment samples and were selected during therapy, possibly because of a lower sensitivity of HBV genotype A to nucleoside nucleotide/analogues. In contrast, Erhardt et al found that genotype A is more sensitive to interferon than genotype D in CHB patients[27]. In this study, we also observed the genotype change from C to B in the majority of 13 genotype shift patients (10/13). One possible explanation for the preferential switch from genotype C to genotype B (rather than from genotype B to genotype C) is that genotype C isolates have lower replication capacity than genotype B and are hence eliminated faster by nucleoside analogues. This idea is supported by an in vitro transfection study [28]. In contrast, 22 patients out of 25 patients without genotype shift were infected dominantly with the genotype B following one year ADV treatment. This observation suggests that genotype C may be more sensitive to ADV-treatment than genotype B. Furthermore, in genotype shift group, genotype B ratio increased significantly accompanied by a decrease of genotype C ratio after ADV therapy. Importantly, patients with dominant genotype C had more significant reduction of ALT and HBeAg after ADV treatment. Therefore, differential drug sensitivity may exist among different genotypes and may be the key factor in determining genotype shift observed in this study. Clinical efficacy of antiviral therapy may be associated with the genotype and genotype shift, both of which are influenced by the external drug selection pressure as exemplified above. Whether the preference in genotype shift is related to the genotype differential drug sensitivity or selection pressure during antiviral therapy remains to be investigated in the future.

In conclusion, we established a new deep sequencing strategy for HBV genotyping during antiviral therapy. For the first time, the rationale of HBV genotype shift has been adequately explained in this study. HBV genotype shifts are associated with the clinical improvement during the drug treatment and may become an important factor for consideration of future antiviral therapy.

## Supporting Information

S1 Fig The workflow of the short-window based HBV genotyping methodology. (TIF)

S2 Fig Establishment of short HBV DNA-based genotyping methodology using deep sequencing. (A) Expected average genotyping accuracy of different window lengths on the consensus HBV genome. The average accuracy of 0.95 or 0.99 could be achieved respectively, when a minimal window length of 75 bp or 200bp were used. (B) Expected genotyping accuracy of 100-bp long window starting from different positions. (C) Comparison of the observed genotype ratios (B & C) of 26 clinical specimens based on clonal sequencing and deep sequencing (GS FLX), respectively. The result indicated a high agreement of C.C. = 0.994 (Table C in S1 File). (D) Comparison of the observed genotype ratios (B & C) of 26 clinical specimens by deep sequencing with GS FLX and Solexa, respectively. The multiple correlation coefficient was C.C. = 0.998.
(TIFF)

S1 File Materials and Methods. (DOC)
