# Supplementary figures and images for: Deep Sequencing Analysis of HBV Genotype Shift and Correlation with Antiviral Efficiency during Adefovir Dipivoxil Therapy
Source: PLoS One. 2015 Jun 25;10(6):e0131337. doi: 10.1371/journal.pone.0131337 (PMC4482366; doi:10.1371/journal.pone.0131337)

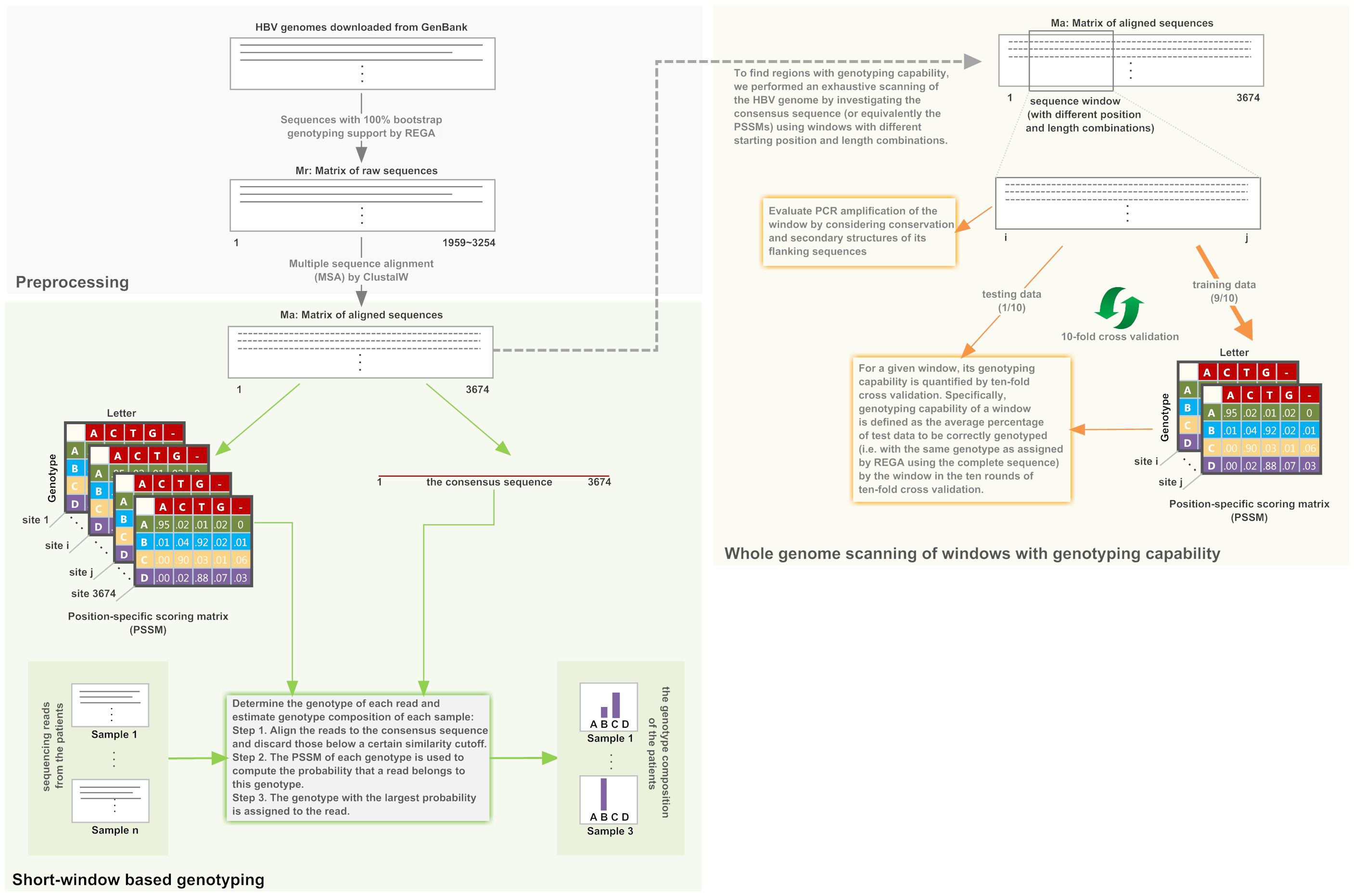

Supplement: S1 Fig — (TIF) [file pone.0131337.s001.tif]

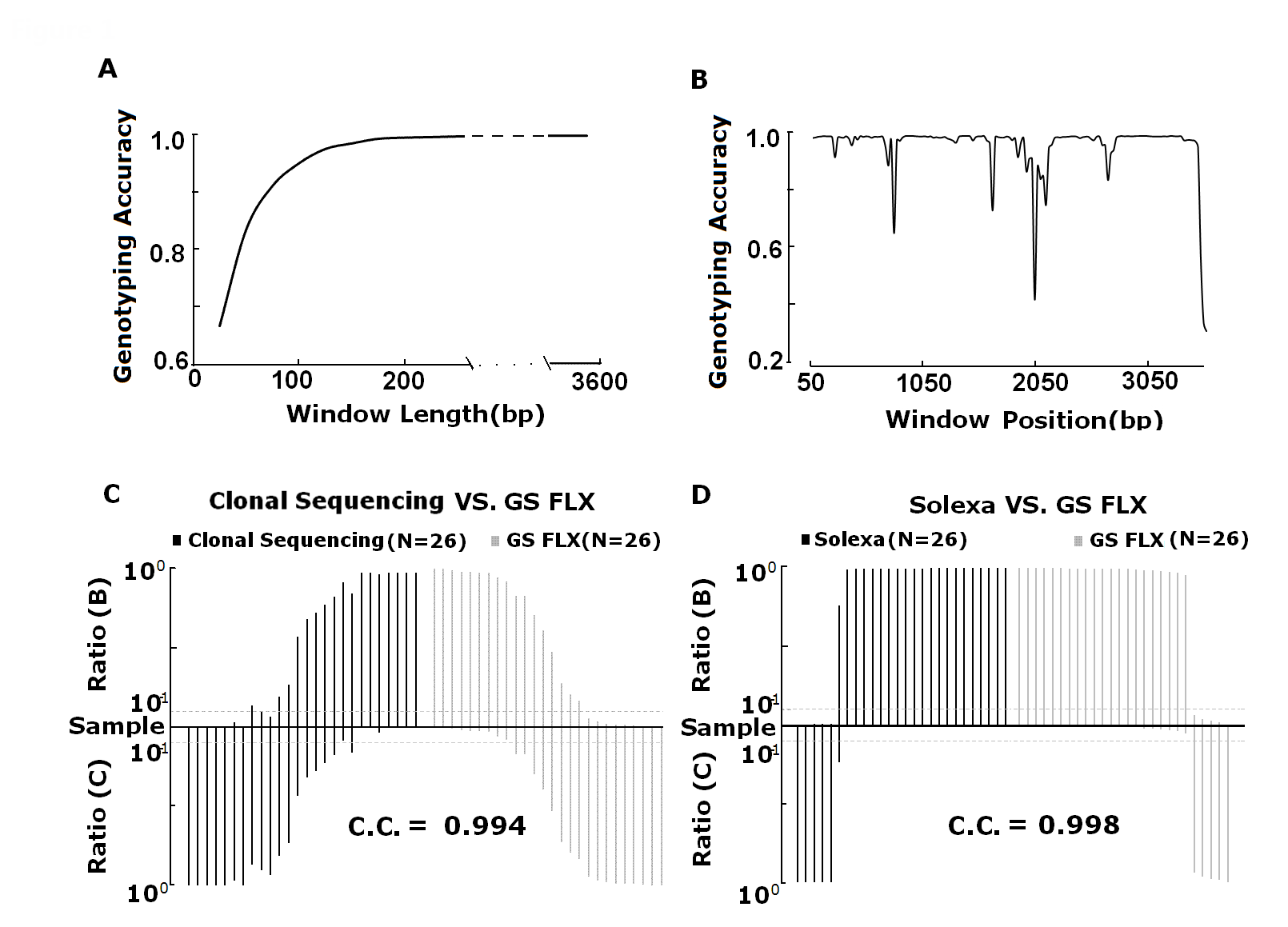

Supplement: S2 Fig — (A) Expected average genotyping accuracy of different window lengths on the consensus HBV genome. The average accuracy of 0.95 or 0.99 could be achieved respectively, when a minimal window length of 75 bp or 200bp were used. (B) Expected genotyping accuracy of 100-bp long window starting from different positions. (C) Comparison of the observed genotype ratios (B & C) of 26 clinical specimens based on clonal sequencing and deep sequencing (GS FLX), respectively. The result indicated a high agreement of C.C. = 0.994 (Table C in S1 File). (D) Comparison of the observed genotype ratios (B & C) of 26 clinical specimens by deep sequencing with GS FLX and Solexa, respectively. The multiple correlation coefficient was C.C. = 0.998. (TIFF) [file pone.0131337.s002.tiff]
