# Supplementary material for: Deep Sequencing Analysis of HBV Genotype Shift and Correlation with Antiviral Efficiency during Adefovir Dipivoxil Therapy
Source: PLoS One. 2015 Jun 25;10(6):e0131337. doi: 10.1371/journal.pone.0131337 (PMC4482366; doi:10.1371/journal.pone.0131337)
Supplement: S1 File — (DOC) [file pone.0131337.s003.doc]

**Materials and Methods**

**Direct and clonal sequencing for HBV genotype analysis**

Serum samples from 38 CHB patients before and after 48 weeks of ADV treatment were used to obtain HBV DNA. PCR amplification was initially performed for the region between nt 599-899bp by using nested-PCR. The primers used were given in Table B. PCR products were performed with direct sequencing by Sanger using 3730 DNA Analyzer (Applied Biosystems, Foster City, CA). Furthermore, 26 selected PCR products obtained by the direct sequencing above were sequenced by clonal analysis to compare the genotyping results with GS FLX deep sequencing.

**Massively parallel deep sequencing for HBV genotyping**

Deep sequencing by GS FLX: Serum samples collected at different time points from 38 ADV-treated patients were subjected to PCR (nt 599-899). The conditions for the first round of PCR amplification was 95°C 3min; 95°C 30s, 55°C 30s, 72°C 45s , 35 cycles and 72°C 5min. For the nested-PCR, the second round was: 95°C 3min; 95°C 30s, 57°C 30s, 72°C 45s, 30 cycles and 72°C 5min. The primers used are given in Table B. Meanwhile, each PCR product was barcoded with an additional tag (8 nucleotides) on both ends. The sequences then can be distributed to the different samples by the specific barcodes. The window of nt 619-879 (261bp) was selected for GS FLX deep sequencing, which has a genotyping accuracy of 100%. GS FLX platform (454 Life Sciences, Roche, Bandford, CT) was used for deep sequencing by Majorbio Bio-Pharm Technology Co., Ltd (Shanghai, China).

Deep sequencing by Solexa: The serum HBV DNA of 200 CHB patients were amplified from the region corresponding to nt 1426-1564 with different sets of primers. The following conditions of PCR amplification were used: 98°C 3min; 95°C 30s, 62°C 30s, 72°C 20s, 35 cycles and 72°C 5min. Meanwhile, each PCR product was barcoded with an additional tag (6-12 nucleotides) on both ends. Therefore, the sequences can be distributed to the different samples by the specific barcodes.The window of nt 1446-1544 (99bp) was selected for Solexa deep sequencing, which has a genotyping accuracy of 99.9%. Deep sequencing with Solexa/Illumina Genome Analyzer method (Illumina Inc, San Diego, CA) was performed by BGI (Shenzhen, China).

**Deep sequencing data analysis**

GS FLX data processing procedure: GS FLX generated 645,248 sequences reads and the median read length was 243 bp（ranged from 24 to 542 bp）. Firstly, those reads which length were lower than 281 bp (the amplified sequences length and primers length were 261 and 20 bp respectively) were eliminated. Secondly, those reads were additionally corrected by an automated computational algorithm that those likely to contain sequencing errors in contiguous poly bases on Poisson distribution. Then, those sequences with an identity score <80% relative to the consensus sequence were also removed. Reads had ambiguous base or stop codon were filtered in the next step. Finally, the average coverage of the remaining sequences was 3,676( ranged from 490 to 8,460).

Solexa data processing procedure: Firstly, the 24,476,895 raw solexa single end reads were distributed to 200 samples by the designed barcodes. Secondly, reads with low quality (phred Q13) bases count were more than 15, or ambiguous bases count were more than 3, or some base proportion of one read was more than 90% were filtered. Then,the reverse sequences were converted to forward. The Position Specific Scoring Matrices (PSSM) was used to evaluate the variation of the sequences, sequences score with lower than 0.7 were filtered. Finally, the average coverage of the remaining sequences was 99,498(ranged from 9,356 to 343,512).

**Validation of genotyping analysis by deep-sequencing**

To verify whether short read length of deep-sequencing can achieve similar genotyping capability as golden standard of HBV genotyping using the whole genome, we performed a comprehensive analysis of the HBV genome by scanning the HBV genome using windows with different starting positions and lengths. An expected genotyping accuracy of 100% can be achieved, when the window length is longer than 250 bp (S2A Fig.). Surprisedly, 100bp window length could still reach an accuracy of 95-100% when the window sites were properly selected (S2B Fig.). These data indicated that a short DNA sequencing-based genotyping strategy could be applied for HBV genotype analysis, and the window sites are the key factor to determine the accuracy of genotyping, especially when the windows length is less than 250bp.

Next, different sequence methods (Clonal sequencing vs. GS FLX and Solexa vs. GS FLX) were also compared to verify genotyping consistency in 13 CHB patients with genotype shift before and after ADV therapy. A correlation coefficient of 0.994 was found in 26 clinical samples between results of clonal sequencing and deep sequencing GS FLX (S2C Fig.). A correlation coefficient of 0.998 was reached when the results of deep sequencing GS FLX were compared with Solexa (S2D Fig.) in 26 clinical specimens. These data indicated that deep sequencing methods are highly comparable to the cloning sequencing, which is generally considered as the golden standard for HBV genotyping.

Furthermore, we performed additional control experiments to identify the accuracy of deep sequencing method for detection of the minor genotype ratio and complexity of quasispecies. 35 samples were prepared by mixing the 8 synthetic sequences at different proportions (S3 and Table Ds) and subjected to Solexa sequencing. The observed ratio correlated positively with theoretical ratio (R^2^=0.994, *P*<2x10^-16^) and the observed sample complexity closely matched the theoretical sample complexity (R^2^=0.991, *P*<2x10^-16^). Of 35 samples, 7 samples showed minor cross contamination and an average cross contamination rate of 0.02% was calculated (Table E) Meanwhile, the normal mouse serum which could not be contaminated with HBV was used as negative control and an average cross contamination rate of 0.02% was obtained. Accordingly, the accuracy of deep sequencing for detecting low-level mixed infection rate was considered to be greater than 0.02%.

The complexity of a sequence mixture is measured by normalized entropy, defined as:

$Complexity=\frac{\sum_{G=1..K} -P_{G}\times log(P_{G})}{\sum_{G=1..K} \frac{log(K)}{K}}$

Where K represents the component number in the mixture, P_G_ represents the fraction of each component. For a system with two components, the maximal complexity is arrived when the two components have the same abundance.

**1. Model building**

The 2,600 HBV genome sequences available from NCBI GenBank on Jun 20, 2011 were downloaded and genotyped using the REGA server (<http://newbioafrica.mrc.ac.za/rega-genotype>). The 1000 sequences with the most reliable genotype assignment (bootstrap support=1) were kept and denoted as SeqSet hereafter, which includes 142 A, 31 B, 752 C, 75 D sequences, respectively. A multiple sequence alignment (MSA) of the sequences in SeqSet was performed using ClustalW. Based on the MAS, two things were done. First, a consensus HBV genome sequence was generated by putting at each site the most frequent letter (A, C, T, G or gap) of the corresponding column of the MSA. Second, for each genotype a position-specific scoring matrix (PSSM) was constructed based on the MSA of the corresponding genotype sequences. Since the consensus sequence and the PSSMs share the same coordinate system derived from the MSA, each element P(B_k_ |T, k) of a PSSM gives the probability for genotype T that the letter B_k_ (A, C, T, G or gap) appears at position k of the consensus sequence. The consensus sequence and the PSSMs have different roles in the process of genotype assignment. To genotype an HBV query sequence, the query is first aligned to the consensus sequence so that the position and span of the query on the consensus sequence or equivalently on the PSSMs can be determined. Then, the query is scored by the PSSM of each genotype and assigned to the genotype with the highest score.

**2. Genotype assignment**

Based on the PSSMs, the genotype of a short sequence fragment of HBV is determined as described at the following. Under the assumption of position independence, the probability of observing a specific sequence of genotype T can be formulated as

$$P(W=B_{W_{1}},B_{W_{2}},...,B_{W_{L}}|T)=\prod_{i=1}^{L} P(B_{W_{i}}|T,W_{i})\text{ }(1)$$

where W represents a L-bp long sequence window starting from position W_1_ to position W_L_.

Applying Bayes’ rule, the posterior probability of genotype T given the sequence W is

$$P(T|W=B_{W_{1}},B_{W_{2}},...,B_{W_{L}})=\frac{P(W=B_{W_{1}},B_{W_{2}},...,B_{W_{L}}|T)P(T)}{P(W=B_{W_{1}},B_{W_{2}},...,B_{W_{L}})}\text{ }(2)$$

The genotype with the greatest posterior probability was assigned to the sequence

$$\mathop{argmax}\limits_{T} P(T|W=B_{W_{1}},B_{W_{2}},...,B_{W_{L}})\text{ }(3)$$

**3. Whole genome scanning of windows with genotyping capability**

To find regions with genotyping capability, we performed an exhaustive scanning of the HBV genome by investigating the consensus sequence using windows with different starting position and length combinations. Given a window, a ten-fold cross-validation was performed to evaluate its genotyping capability or reliability. Concretely, the SeqSet was split into ten approximately equal partitions. Each in turn is used for testing and the remainder is used for training. That is, use of the data to build PSSMs of different HBV genotypes and the remainder to perform genotyping based on the constructed PSSMs according to equation (3). The genotyping capability of the window is calculated as the proportion of sequences in the holdout set with consistent genotype assignment as REGA. The procedure was repeated for ten times and the ten genotyping capability estimates are averaged to yield an overall estimate.

**4. Short-window based genotyping**

To determine the genotype of a query sequence, for instance from the reads of a deep sequencing experiment, the following procedures are followed. First, the query is aligned to the consensus HBV genome sequence and discarded if it is below a certain similarity cutoff, which is determined using non-HBV sequences. More concretely, the query is aligned to the consensus sequence twice, once in its original form, the other in the reverse order. If both alignments are below the similarity cutoff, the read is regarded as a non-HBV sequence and discarded. Otherwise, the alignment with the highest degree of match is used to determine the final position and span of the query on the consensus HBV genome or equivalently on the PSSMs. Then, the PSSM of each genotype is used to compute the likelihood that the read belongs to this genotype (equation 2). Finally, the genotype with the largest likelihood is assigned to the read (equation 3).

**Table A** Characteristics of the 38 ADV-treated chronic hepatitis B patients.

**Table B**  List of primers used for the deep sequencing GS FLX

**Table C** List of DNA sequences of eight synthetic oligo nucleotides (139bp)

**Table D** List of 35 combinations with different ratios of synthetic DNA sequences

**Table E** 280 individual template ratios (theoretical Vs observed) out of 35 combinations of different synthetic sequences

**Table A**

Characteristics of the 38 ADV-treated chronic hepatitis B patients

| **Features** | **Description** | ***p*-value** |
| --- | --- | --- |
| Age | 30.3 (19-52) |  |
| Sex(male/female) | 32/6 |  |
| Alanine aminotransferase(IU/mL) |  | 0.026 |
| Pre-treatment | 161.8(45-611) |  |
| After treatment | 65.1 (16-451) |  |
| HBV DNA (log_10_copies/mL) |  | 0.046 |
| Pre-treatment | 9.5 (7.3-11.1) |  |
| After treatment | 7.3 (3.0-10.6) |  |
| Hepatitis B e antigen (+/-) |  | 0.024 |
| Pre-treatment | 38 / 0 |  |
| After treatment | 20 /18 |  |

**Table B**

List of primers used for the deep sequencing GS FLX

| **Sample** | **Forward primer** | **Reverse primer** |
| --- | --- | --- |
| 1B | 5' - AAACTGAG -TGTATTCCCATCCCATCATC-3' | 5'-AAACTGAG-CAAC(T/C/A)(T/C)CCAATTACATATCC-3' |
| 1A | 5' - CATATGAG -TGTATTCCCATCCCATCATC-3' | 5'- CATATGAG -CAAC(T/C/A)(T/C)CCAATTACATATCC-3' |
| 2B | 5' - AGACTCAT-TGTATTCCCATCCCATCATC-3' | 5'- AGACTCAT-CAAC(T/C/A)(T/C)CCAATTACATATCC-3' |
| 2A | 5' - CACTAGCT-TGTATTCCCATCCCATCATC-3' | 5'- CACTAGCT-CAAC(T/C/A)(T/C)CCAATTACATATCC-3' |
| 3B | 5' -CTCAGCAT-TGTATTCCCATCCCATCATC-3' | 5'- CTCAGCAT-CAAC(T/C/A)(T/C)CCAATTACATATCC-3' |
| 3A | 5' - TATCTATG -TGTATTCCCATCCCATCATC-3' | 5'- TATCTATG -CAAC(T/C/A)(T/C)CCAATTACATATCC-3' |
| 4B | 5' - ACGCATAT-TGTATTCCCATCCCATCATC-3' | 5'- ACGCATAT-CAAC(T/C/A)(T/C)CCAATTACATATCC-3' |
| 4A | 5' - CAGCTAGT-TGTATTCCCATCCCATCATC-3' | 5'- CAGCTAGT-CAAC(T/C/A)(T/C)CCAATTACATATCC-3' |
| 5B | 5' - agagacat -TGTATTCCCATCCCATCATC-3' | 5'- agagacat-CAAC(T/C/A)(T/C)CCAATTACATATCC-3' |
| 5A | 5' -agtacgag-TGTATTCCCATCCCATCATC-3' | 5'-agtacgag-CAAC(T/C/A)(T/C)CCAATTACATATCC-3' |
| 6B | 5' - ATAGTACG-TGTATTCCCATCCCATCATC-3' | 5'- ATAGTACG-CAAC(T/C/A)(T/C)CCAATTACATATCC-3' |
| 6A | 5' - ATACATAT-TGTATTCCCATCCCATCATC-3' | 5'- ATACATAT-CAAC(T/C/A)(T/C)CCAATTACATATCC-3' |
| 7B | 5' - TAGACAGT-TGTATTCCCATCCCATCATC-3' | 5'- TAGACAGT-CAAC(T/C/A)(T/C)CCAATTACATATCC-3' |
| 7A | 5' - TGAGAGCG -TGTATTCCCATCCCATCATC-3' | 5'- TGAGAGCG -CAAC(T/C/A)(T/C)CCAATTACATATCC-3' |
| 8B | 5' - TAGACATG-TGTATTCCCATCCCATCATC-3' | 5'- TAGACATG-CAAC(T/C/A)(T/C)CCAATTACATATCC-3' |
| 8A | 5' - ACTCAGTG -TGTATTCCCATCCCATCATC-3' | 5'- ACTCAGTG -CAAC(T/C/A)(T/C)CCAATTACATATCC-3' |
| 9B | 5' - ATACTGTG-TGTATTCCCATCCCATCATC-3' | 5'- ATACTGTG-CAAC(T/C/A)(T/C)CCAATTACATATCC-3' |
| 9A | 5' - ATGACTAT-TGTATTCCCATCCCATCATC-3' | 5'- ATGACTAT-CAAC(T/C/A)(T/C)CCAATTACATATCC-3' |
| 10B | 5' - ACGTATCG-TGTATTCCCATCCCATCATC-3 | 5'- ACGTATCG-CAAC(T/C/A)(T/C)CCAATTACATATCC-3' |
| 10A | 5' - ATCTCAGT-TGTATTCCCATCCCATCATC-3' | 5'- ATCTCAGT-CAAC(T/C/A)(T/C)CCAATTACATATCC-3' |
| 11B | 5' -agtgctcg-TGTATTCCCATCCCATCATC-3' | 5'-agtgctcg-CAAC(T/C/A)(T/C)CCAATTACATATCC-3' |
| 11A | 5' -acagctat-TGTATTCCCATCCCATCATC-3' | 5'-acagctat-CAAC(T/C/A)(T/C)CCAATTACATATCC-3' |
| 12B | 5' - CAGTGCGT-TGTATTCCCATCCCATCATC-3' | 5'- CAGTGCGT-CAAC(T/C/A)(T/C)CCAATTACATATCC-3' |
| 12A | 5' - CACACTCT-TGTATTCCCATCCCATCATC-3' | 5'- CACACTCT-CAAC(T/C/A)(T/C)CCAATTACATATCC-3' |
| 13B | 5' - TACGACTG-TGTATTCCCATCCCATCATC-3' | 5'- TACGACTG-CAAC(T/C/A)(T/C)CCAATTACATATCC-3' |
| 13A | 5' - ACTCATCT -TGTATTCCCATCCCATCATC-3' | 5'- ACTCATCT -CAAC(T/C/A)(T/C)CCAATTACATATCC-3' |
| 14B | 5' - TATAGCAT-TGTATTCCCATCCCATCATC-3' | 5'- TATAGCAT-CAAC(T/C/A)(T/C)CCAATTACATATCC-3' |
| 14A | 5' - ACTCTACG -TGTATTCCCATCCCATCATC-3' | 5'- ACTCTACG -CAAC(T/C/A)(T/C)CCAATTACATATCC-3' |
| 15B | 5' - CATGACAT-TGTATTCCCATCCCATCATC-3' | 5'- CATGACAT-CAAC(T/C/A)(T/C)CCAATTACATATCC-3' |
| 15A | 5' - AGCTAGTG-TGTATTCCCATCCCATCATC-3' | 5'- AGCTAGTG-CAAC(T/C/A)(T/C)CCAATTACATATCC-3' |
| 16B | 5' - TATAGTAG-TGTATTCCCATCCCATCATC-3' | 5'- TATAGTAG-CAAC(T/C/A)(T/C)CCAATTACATATCC-3' |
| 16A | 5' - ATAGAGTG -TGTATTCCCATCCCATCATC-3' | 5'- ATAGAGTG -CAAC(T/C/A)(T/C)CCAATTACATATCC-3' |
| 17B | 5' - TATAGTGT-TGTATTCCCATCCCATCATC-3' | 5'- TATAGTGT-CAAC(T/C/A)(T/C)CCAATTACATATCC-3' |
| 17A | 5' - TGACAGAG -TGTATTCCCATCCCATCATC-3' | 5'- TGACAGAG -CAAC(T/C/A)(T/C)CCAATTACATATCC-3' |
| 18B | 5' - CTAGTACT-TGTATTCCCATCCCATCATC-3' | 5'- CTAGTACT-CAAC(T/C/A)(T/C)CCAATTACATATCC-3' |
| 18A | 5' - CTAGTCAG-TGTATTCCCATCCCATCATC-3' | 5'- CTAGTCAG-CAAC(T/C/A)(T/C)CCAATTACATATCC-3' |
| 19B | 5' - TATATCTG-TGTATTCCCATCCCATCATC-3' | 5'- TATATCTG-CAAC(T/C/A)(T/C)CCAATTACATATCC-3' |
| 19A | 5' - ATAGACGT -TGTATTCCCATCCCATCATC-3' | 5'- ATAGACGT -CAAC(T/C/A)(T/C)CCAATTACATATCC-3' |
| 20B | 5' - TATGAGCG-TGTATTCCCATCCCATCATC-3' | 5'- TATGAGCG-CAAC(T/C/A)(T/C)CCAATTACATATCC-3' |
| 20A | 5' - TGTGCACG -TGTATTCCCATCCCATCATC-3' | 5'- TGTGCACG -CAAC(T/C/A)(T/C)CCAATTACATATCC-3' |
| 21B | 5' - CACTCTGT -TGTATTCCCATCCCATCATC-3' | 5'- CACTCTGT -CAAC(T/C/A)(T/C)CCAATTACATATCC-3' |
| 21A | 5' - TATCATGT-TGTATTCCCATCCCATCATC-3' | 5'- TATCATGT-CAAC(T/C/A)(T/C)CCAATTACATATCC-3' |
| 22B | 5' - ACACACAT-TGTATTCCCATCCCATCATC-3' | 5'-ACACACAT-CAAC(T/C/A)(T/C)CCAATTACATATCC-3' |
| 22A | 5' - ACATATAG-TGTATTCCCATCCCATCATC-3' | 5'- ACATATAG -CAAC(T/C/A)(T/C)CCAATTACATATCC-3' |
| 23B | 5' - ACGAGCAT -TGTATTCCCATCCCATCATC-3' | 5'-ACGAGCAT-CAAC(T/C/A)(T/C)CCAATTACATATCC-3' |
| 23A | 5' - ATCTATCT -TGTATTCCCATCCCATCATC-3' | 5'- ATCTATCT -CAAC(T/C/A)(T/C)CCAATTACATATCC-3' |
| 24B | 5' - ATACGTCG-TGTATTCCCATCCCATCATC-3' | 5'- ATACGTCG-CAAC(T/C/A)(T/C)CCAATTACATATCC-3' |
| 24A | 5' - ATACATCG-TGTATTCCCATCCCATCATC-3' | 5'- ATACATCG-CAAC(T/C/A)(T/C)CCAATTACATATCC-3' |
| 25B | 5' -ATACACAG -TGTATTCCCATCCCATCATC-3' | 5'-ATACACAG -CAAC(T/C/A)(T/C)CCAATTACATATCC-3' |
| 25A | 5' -ATACGACG-TGTATTCCCATCCCATCATC-3' | 5'-ATACGACG-CAAC(T/C/A)(T/C)CCAATTACATATCC-3' |
| 26B | 5'-ATATAGCT-TGTATTCCCATCCCATCATC-3' | 5'-ATATAGCT-CAAC(T/C/A)(T/C)CCAATTACATATCC-3' |
| 26A | 5'-ATGCACGT-TGTATTCCCATCCCATCATC-3' | 5'-ATGCACGT-CAAC(T/C/A)(T/C)CCAATTACATATCC-3' |
| 27B | 5' - ACGTATAT-TGTATTCCCATCCCATCATC-3' | 5'- ACGTATAT-CAAC(T/C/A)(T/C)CCAATTACATATCC-3' |
| 27A | 5' - ACGAGCGT-TGTATTCCCATCCCATCATC-3' | 5'- ACGAGCGT-CAAC(T/C/A)(T/C)CCAATTACATATCC-3' |
| 28B | 5' - ACACGACT-TGTATTCCCATCCCATCATC-3' | 5'- ACACGACT-CAAC(T/C/A)(T/C)CCAATTACATATCC-3' |
| 28A | 5' - ACAGTGCG-TGTATTCCCATCCCATCATC-3' | 5'- ACAGTGCG -CAAC(T/C/A)(T/C)CCAATTACATATCC-3' |
| 29B | 5' -ATCATCAT-TGTATTCCCATCCCATCATC-3' | 5'-ATCATCAT-CAAC(T/C/A)(T/C)CCAATTACATATCC-3' |
| 29A | 5' - ATCGAGCT-TGTATTCCCATCCCATCATC-3' | 5'- ATCGAGCT-CAAC(T/C/A)(T/C)CCAATTACATATCC-3' |
| 30B | 5' - ATCTCATG-TGTATTCCCATCCCATCATC-3' | 5'- ATCTCATG-CAAC(T/C/A)(T/C)CCAATTACATATCC-3' |
| 30A | 5' - ATATGACT-TGTATTCCCATCCCATCATC-3' | 5'- ATATGACT-CAAC(T/C/A)(T/C)CCAATTACATATCC-3' |
| 31B | 5' - AGTGTGAG -TGTATTCCCATCCCATCATC-3' | 5'- AGTGTGAG -CAAC(T/C/A)(T/C)CCAATTACATATCC-3' |
| 31A | 5' - AGTATCAT -TGTATTCCCATCCCATCATC-3' | 5'- AGTATCAT -CAAC(T/C/A)(T/C)CCAATTACATATCC-3' |
| 32B | 5' - ATAGCGCT -TGTATTCCCATCCCATCATC-3' | 5'- ATAGCGCT -CAAC(T/C/A)(T/C)CCAATTACATATCC-3' |
| 32A | 5' - ATAGCTGT -TGTATTCCCATCCCATCATC-3' | 5'- ATAGCTGT -CAAC(T/C/A)(T/C)CCAATTACATATCC-3' |
| 33B | 5' - AGAGCGAG-TGTATTCCCATCCCATCATC-3' | 5'- AGAGCGAG-CAAC(T/C/A)(T/C)CCAATTACATATCC-3' |
| 33A | 5' - ATAGACTG-TGTATTCCCATCCCATCATC-3' | 5'- ATAGACTG-CAAC(T/C/A)(T/C)CCAATTACATATCC-3' |
| 34B | 5' - CATCGTAT-TGTATTCCCATCCCATCATC-3' | 5'- CATCGTAT-CAAC(T/C/A)(T/C)CCAATTACATATCC-3' |
| 34A | 5' - AGAGCGTG-TGTATTCCCATCCCATCATC-3' | 5'-AGAGCGTG-CAAC(T/C/A)(T/C)CCAATTACATATCC-3' |
| 35B | 5' - ACTCGTCT-TGTATTCCCATCCCATCATC-3' | 5'- ACTCGTCT-CAAC(T/C/A)(T/C)CCAATTACATATCC-3' |
| 35A | 5' - ACTCATAT-TGTATTCCCATCCCATCATC-3' | 5'- ACTCATAT-CAAC(T/C/A)(T/C)CCAATTACATATCC-3' |
| 36B | 5' - AGACATAG-TGTATTCCCATCCCATCATC-3' | 5'- AGACATAG-CAAC(T/C/A)(T/C)CCAATTACATATCC-3' |
| 36A | 5' - AGTACATG-TGTATTCCCATCCCATCATC-3' | 5'-AGTACATG-CAAC(T/C/A)(T/C)CCAATTACATATCC-3' |
| 37B | 5' - AGTGACAG-TGTATTCCCATCCCATCATC-3' | 5'-AGTGACAG-CAAC(T/C/A)(T/C)CCAATTACATATCC-3' |
| 37A | 5' - AGTGTACG-TGTATTCCCATCCCATCATC-3' | 5'- AGTGTACG-CAAC(T/C/A)(T/C)CCAATTACATATCC-3' |
| 38B | 5' - AGTCAGCT-TGTATTCCCATCCCATCATC-3' | 5'-AGTCAGCT-CAAC(T/C/A)(T/C)CCAATTACATATCC-3' |
| 38A | 5' - AGTCATCG-TGTATTCCCATCCCATCATC-3' | 5'-AGTCATCG-CAAC(T/C/A)(T/C)CCAATTACATATCC-3' |

A refers to after treatment, B refers to before treatment. The underlined sequences indicate barcode tags.

**Table C**

List of DNA sequences of eight synthetic oligo nucleotides (139bp)

| **ID** | **Sequence** |
| --- | --- |
| 1 | TACGTCCCGTCGGCGCTGAA-**AC**CCGCGGACGACCCGTCTCGGGGC**AC**TTTGGGACTCTACCGTCCCCTT**AC**TCATCTGCCGTTCCGGCCAACC**AC**GGGGCGCACCTCTCTTTACGCG**AC**-CTCCCCGTCTGTGCCTTCTC |
| 2 | TACGTCCCGTCGGCGCTGAA-**AG**CCGCGGACGACCCGTCTCGGGGC**AG**TTTGGGACTCTACCGTCCCCTT**AG**TCATCTGCCGTTCCGGCCAACC**AG**GGGGCGCACCTCTCTTTACGCG**AG**-CTCCCCGTCTGTGCCTTCTC |
| 3 | TACGTCCCGTCGGCGCTGAA-**CT**CCGCGGACGACCCGTCTCGGGGC**CT**TTTGGGA  CTCTACCGTCCCCTT**CT**TCATCTGCCGTTCCGGCCAACC**CT**GGGGCGCACCTCTCTTTACGCG**CT**-CTCCCCGTCTGTGCCTTCTC |
| 4 | TACGTCCCGTCGGCGCTGAA-**CA**CCGCGGACGACCCGTCTCGGGGC**CA**TTTGGGACTCTACCGTCCCCTT**CA**TCATCTGCCGTTCCGGCCAACC**CA**GGGGCGCACCTCTCTTTACGCG**CA**-CTCCCCGTCTGTGCCTTCTC |
| 5 | TACGTCCCGTCGGCGCTGAA-**TC**CCGCGGACGACCCGTCTCGGGGC**TC**TTTGGGA  CTCTACCGTCCCCTT**TC**TCATCTGCCGTTCCGGCCAACC**TC**GGGGCGCACCTCTCTTTACGCG**TC**-CTCCCCGTCTGTGCCTTCTC |
| 6 | TACGTCCCGTCGGCGCTGAA-**TG**CCGCGGACGACCCGTCTCGGGGC**TG**TTTGGGACTCTACCGTCCCCTT**TG**TCATCTGCCGTTCCGGCCAACC**TG**GGGGCGCACCTCTCTTTACGCG**TG**-CTCCCCGTCTGTGCCTTCTC |
| 7 | TACGTCCCGTCGGCGCTGAA-**GT**CCGCGGACGACCCGTCTCGGGGC**GT**TTTGGGACTCTACCGTCCCCTT**GT**TCATCTGCCGTTCCGGCCAACCGTGGGGCGCACCTCTCTTTACGCG**GT**-CTCCCCGTCTGTGCCTTCTC |
| 8 | TACGTCCCGTCGGCGCTGAA-**GA**CCGCGGACGACCCGTCTCGGGGC**GA**TTTGGGACTCTACCGTCCCCTT**GA**TCATCTGCCGTTCCGGCCAACC**GA**GGGGCGCACCTCTCTTTACGCG**GA**-CTCCCCGTCTGTGCCTTCTC |

The underlined sequences are forward and reverse primers. The bold letters indicate mutation nucleotides.

**Table D**

List of 35 combinations with different ratios of synthetic DNA sequences

| **combination** | **^*^S1** | **S2** | **S3** | **S4** | **S5** | **S6** | **S7** | **S8** |
| --- | --- | --- | --- | --- | --- | --- | --- | --- |
| 1 | 0.99 | 0.01 |  |  |  |  |  |  |
| 2 | 0.96 | 0.04 |  |  |  |  |  |  |
| 3 | 0.92 | 0.08 |  |  |  |  |  |  |
| 4 | 0.87 | 0.13 |  |  |  |  |  |  |
| 5 | 0.8 | 0.2 |  |  |  |  |  |  |
| 6 | 0.7 | 0.3 |  |  |  |  |  |  |
| 7 | 0.5 | 0.5 |  |  |  |  |  |  |
| 8 | 0.99 | 0.003 | 0.003 | 0.004 |  |  |  |  |
| 9 | 0.98 | 0.01 | 0.005 | 0.005 |  |  |  |  |
| 10 | 0.96 | 0.013 | 0.013 | 0.014 |  |  |  |  |
| 11 | 0.92 | 0.04 | 0.02 | 0.02 |  |  |  |  |
| 12 | 0.85 | 0.07 | 0.04 | 0.04 |  |  |  |  |
| 13 | 0.495 | 0.005 | 0.495 | 0.005 |  |  |  |  |
| 14 | 0.77 | 0.12 | 0.06 | 0.05 |  |  |  |  |
| 15 | 0.67 | 0.17 | 0.08 | 0.08 |  |  |  |  |
| 16 | 0.435 | 0.065 | 0.435 | 0.065 |  |  |  |  |
| 17 | 0.54 | 0.23 | 0.12 | 0.11 |  |  |  |  |
| 18 | 0.33 | 0.33 | 0.16 | 0.17 |  |  |  |  |
| 19 | 0.25 | 0.25 | 0.25 | 0.25 |  |  |  |  |
| 20 | 0.5 | 0.17 | 0.17 | 0.16 |  |  |  |  |
| 21 | 0.4 | 0.2 | 0.4 | 0.2 |  |  |  |  |
| 22 | 0.97 | 0.01 | 0.005 | 0.005 | 0.0025 | 0.0025 | 0.0025 | 0.0025 |
| 23 | 0.94 | 0.02 | 0.01 | 0.01 | 0.005 | 0.005 | 0.005 | 0.005 |
| 24 | 0.92 | 0.03 | 0.02 | 0.01 | 0.005 | 0.005 | 0.005 | 0.005 |
| 25 | 0.88 | 0.04 | 0.02 | 0.02 | 0.01 | 0.01 | 0.01 | 0.01 |
| 26 | 0.84 | 0.05 | 0.02 | 0.03 | 0.015 | 0.015 | 0.015 | 0.015 |
| 27 | 0.79 | 0.07 | 0.03 | 0.04 | 0.02 | 0.02 | 0.02 | 0.01 |
| 28 | 0.7 | 0.1 | 0.05 | 0.05 | 0.03 | 0.03 | 0.02 | 0.02 |
| 29 | 0.65 | 0.12 | 0.06 | 0.06 | 0.02 | 0.03 | 0.03 | 0.03 |
| 30 | 0.57 | 0.14 | 0.07 | 0.07 | 0.04 | 0.04 | 0.03 | 0.03 |
| 31 | 0.5 | 0.17 | 0.08 | 0.09 | 0.04 | 0.04 | 0.04 | 0.04 |
| 32 | 0.44 | 0.19 | 0.1 | 0.09 | 0.05 | 0.05 | 0.04 | 0.04 |
| 33 | 0.33 | 0.22 | 0.11 | 0.11 | 0.05 | 0.06 | 0.06 | 0.06 |
| 34 | 0.25 | 0.25 | 0.12 | 0.13 | 0.06 | 0.06 | 0.06 | 0.07 |
| 35 | 0.125 | 0.125 | 0.125 | 0.125 | 0.125 | 0.125 | 0.125 | 0.125 |

^*^S1 to S8 represents 8 different synthetic DNA sequences listed in Table C

**Table E**

280 individual template ratios (theoretical Vs observed) out of 35 combinations of different synthetic sequences

|  | **^*^S1** | **S2** | **S3** | **S4** | **S5** | **S6** | **S7** | **S8** |
| --- | --- | --- | --- | --- | --- | --- | --- | --- |
| **combination** | **Theoretical Observed** | **Theoretical Observed** | **Theoretical Observed** | **Theoretical Observed** | **Theoretical Observed** | **Theoretical Observed** | **Theoretical Observed** | **Theoretical Observed** |
| 1 | 0.99  0.9827 | 0.01  0.0171 | 0  0 | 0  0 | 0  0 | 0  0 | 0  0 | 0  0 |
| 2 | 0.96  0.9404 | 0.04  0.0595 | 0  0 | 0  0 | 0  0 | 0  0 | 0  0 | 0  0 |
| 3 | 0.92  0.9023 | 0.08  0.0975 | 0  0 | 0  0 | 0  0 | 0  0 | 0  0 | 0  0 |
| 4 | 0.87  0.8179 | 0.13  0.182 | 0  0 | 0  0 | 0  0 | 0  0 | 0  0 | 0  0 |
| 5 | 0.8  0.7316 | 0.2  0.2682 | 0  0 | 0  0 | 0  0 | 0  0 | 0  0 | 0  0 |
| 6 | 0.7  0.6347 | 0.3  0.3652 | 0  0 | 0  0 | 0  0 | 0  0 | 0  0 | 0  0 |
| 7 | 0.5  0.4928 | 0.5  0.5071 | 0  0 | 0  0 | 0  0 | 0  0 | 0  0 | 0  0 |
| 8 | 0.99  0.9956 | 0.003  0.0007 | 0.003  0.0015 | 0.004  0.0019 | 0  0 | 0  0 | 0  0 | 0  0 |
| 9 | 0.98  0.976 | 0.01  0.0178 | 0.005  0.003 | 0.005  0.0029 | 0  0 | 0  0 | 0  0 | 0  0 |
| 10 | 0.96  0.9191 | 0.013  0.0217 | 0.013  0.0259 | 0.014  0.0331 | 0  0 | 0  0 | 0  0 | 0  0 |
| 11 | 0.92  0.8546 | 0.04  0.0591 | 0.02  0.0406 | 0.02  0.0455 | 0  0 | 0  0 | 0  0 | 0  0 |
| 12 | 0.85  0.8016 | 0.07  0.0789 | 0.04  0.0575 | 0.04  0.0618 | 0  0 | 0  0 | 0  0 | 0  0 |
| 13 | 0.495  0.4906 | 0.005  0.0005 | 0.495  0.5076 | 0.005  0.001 | 0  0 | 0  0 | 0  0 | 0  0 |
| 14 | 0.77  0.6783 | 0.12  0.1761 | 0.06  0.0751 | 0.05  0.0701 | 0  0 | 0  0 | 0  0 | 0  0 |
| 15 | 0.67  0.5993 | 0.17  0.214 | 0.08  0.0889 | 0.08  0.0974 | 0  0 | 0  0 | 0  0 | 0  0.0001 |
| 16 | 0.435  0.4276 | 0.065  0.07 | 0.435  0.4348 | 0.065  0.0672 | 0  0 | 0  0 | 0  0 | 0  0.0001 |
| 17 | 0.54  0.457 | 0.23  0.2474 | 0.12  0.1473 | 0.11  0.1479 | 0  0 | 0  0 | 0  0 | 0  0.0001 |
| 18 | 0.33  0.3063 | 0.33  0.3314 | 0.16  0.1704 | 0.17  0.1915 | 0  0 | 0  0 | 0  0 | 0  0.0003 |
| 19 | 0.25  0.2408 | 0.25  0.2503 | 0.25  0.2442 | 0.25  0.2642 | 0  0 | 0  0 | 0  0 | 0  0.0003 |
| 20 | 0.5  0.4217 | 0.17  0.1928 | 0.17  0.1873 | 0.16  0.1976 | 0  0 | 0  0 | 0  0 | 0  0.0003 |
| 21 | 0.4  0.3157 | 0.2  0.1903 | 0.4  0.3121 | 0.2  0.1814 | 0  0 | 0  0 | 0  0 | 0  0.0003 |
| 22 | 0.97  0.9749 | 0.01  0.0145 | 0.005  0.0017 | 0.005  0.0021 | 0.0025  0.0011 | 0.0025  0.0016 | 0.0025  0.0019 | 0.0025  0.0019 |
| 23 | 0.94  0.921 | 0.02  0.0293 | 0.01  0.017 | 0.01  0.0194 | 0.005  0.0021 | 0.005  0.0036 | 0.005  0.0033 | 0.005  0.004 |
| 24 | 0.92  0.8989 | 0.03  0.0412 | 0.02  0.0302 | 0.01  0.0183 | 0.005  0.0019 | 0.005  0.0029 | 0.005  0.0028 | 0.005  0.0033 |
| 25 | 0.88  0.814 | 0.04  0.0437 | 0.02  0.0269 | 0.02  0.0291 | 0.01  0.0168 | 0.01  0.0243 | 0.01  0.0219 | 0.01  0.0229 |
| 26 | 0.84  0.7413 | 0.05  0.0574 | 0.02  0.0268 | 0.03  0.0453 | 0.015  0.0255 | 0.015  0.0371 | 0.015  0.032 | 0.015  0.0343 |
| 27 | 0.79  0.7033 | 0.07  0.0696 | 0.03  0.0352 | 0.04  0.0545 | 0.02  0.0337 | 0.02  0.0429 | 0.02  0.0374 | 0.01  0.023 |
| 28 | 0.7  0.5959 | 0.1  0.1252 | 0.05  0.0505 | 0.05  0.0584 | 0.03  0.0425 | 0.03  0.0554 | 0.02  0.0329 | 0.02  0.0387 |
| 29 | 0.65  0.5751 | 0.12  0.1317 | 0.06  0.0561 | 0.06  0.0623 | 0.02  0.0314 | 0.03  0.0479 | 0.03  0.0463 | 0.03  0.0489 |
| 30 | 0.57  0.5265 | 0.14  0.1382 | 0.07  0.0629 | 0.07  0.0681 | 0.04  0.0533 | 0.04  0.0621 | 0.03  0.0422 | 0.03  0.0463 |
| 31 | 0.5  0.4387 | 0.17  0.1628 | 0.08  0.0694 | 0.09  0.0869 | 0.04  0.0596 | 0.04  0.0634 | 0.04  0.0566 | 0.04  0.0621 |
| 32 | 0.44  0.3729 | 0.19  0.1787 | 0.1  0.1072 | 0.09  0.084 | 0.05  0.0692 | 0.05  0.0712 | 0.04  0.0565 | 0.04  0.0598 |
| 33 | 0.33  0.2699 | 0.22  0.1982 | 0.11  0.1035 | 0.11  0.1197 | 0.05  0.0643 | 0.06  0.0835 | 0.06  0.0758 | 0.06  0.0848 |
| 34 | 0.25  0.212 | 0.25  0.2209 | 0.12  0.1131 | 0.13  0.1335 | 0.06  0.0699 | 0.06  0.0827 | 0.06  0.0738 | 0.07  0.0936 |
| 35 | 0.125  0.1102 | 0.125  0.1125 | 0.125  0.1102 | 0.125  0.1362 | 0.125  0.1367 | 0.125  0.1374 | 0.125  0.111 | 0.125  0.1454 |

^*^S1 to S8 represents 8 different synthetic DNA sequences listed in Table C. There are 7 cross contaminated samples and the average cross contamination rate was 0.02% .
